# Concurrent acute Vogt-Koyanagi-Harada disease in one eye and chronic disease in the fellow eye

**DOI:** 10.1186/s12348-015-0057-9

**Published:** 2015-09-03

**Authors:** Sana Khochtali, Nesrine Abroug, Mohamed Salah Hani, Imen Khairallah-Ksiaa, Bechir Jelliti, Moncef Khairallah

**Affiliations:** Department of Ophthalmology, Fattouma Bourguiba University Hospital, Faculty of Medicine, University of Monastir, Monastir, Tunisia

**Keywords:** Vogt-Koyanagi-Harada disease, Exudative retinal detachment, Sunset-glow fundus

## Abstract

**Background:**

Vogt-Koyanagi-Harada (VKH) disease is a granulomatous panuveitis, usually involving both eyes at the same time or within a few days or weeks. Acute and chronic diseases are characterized by distinct clinical features, treatment modalities, and visual outcomes. We report an atypical case of probable VKH disease, with features of acute disease in one eye and chronic disease in the fellow eye.

**Findings:**

A 53-year-old female presented with exudative retinal detachment associated with mild vitritis in the right eye and anterior uveitis, vitritis, and sunset-glow fundus in the left eye. Based on clinical findings and results of multimodal imaging including fundus photography, spectral-domain optical coherence tomography, fluorescein angiography, indocyanine green angiography, and B-scan ultrasonography, a diagnosis of acute VKH disease in the right eye and chronic VKH disease in the left eye was made. The patient received systemic corticosteroids and mycophenolate mofetil. After a 15-month follow-up, the right fundus eye was normal, and there was a sunset-glow fundus in the left eye.

**Conclusions:**

VKH disease may begin with asymptomatic unilateral ocular involvement. The patient may present only when the fellow eye is affected. A significant delay before involvement of the second eye leads to atypical features of acute disease in one eye and chronic disease in the other eye.

## Findings

### Introduction

Vogt-Koyanagi-Harada (VKH) disease is often defined as a bilateral, chronic granulomatous panuveitis associated with central nervous system, auditory, and integumentary manifestations. The diagnosis is established on the basis of the Revised Diagnostic Criteria for VKH disease [1]. The disease is bilateral and naturally evolves through four consecutive stages. The prodromal stage including neurological and auditory manifestations is followed after few days by the acute uveitic stage. Acute VKH disease typically presents in the form of localized or multifocal exudative retinal detachment (ERD) associated with intraocular inflammatory reaction. It usually affects both eyes at the same time. However, onset may be unilateral, and the second eye may become involved 2 to 4 weeks later [1–3]. The chronic convalescent stage occurs several weeks after the acute stage and is characterized by the development of ocular depigmentation, the sunset-glow fundus being the most specific feature, associated or not with integumentary depigmentation. The chronic recurrent stage usually manifests with granulomatous anterior uveitis and is associated with higher risks of complications and vision loss [2, 3].

We report herein an atypical case of probable VKH disease with concurrent findings of acute disease in one eye and chronic disease in the fellow eye, at first presentation.

### Case report

A 53-year-old female with a history of arterial hypertension presented to our department, with bilateral vision blurring for 5 days. She reported no previous ocular complaints or neurological or auditory symptoms.

**Fig. 1:**
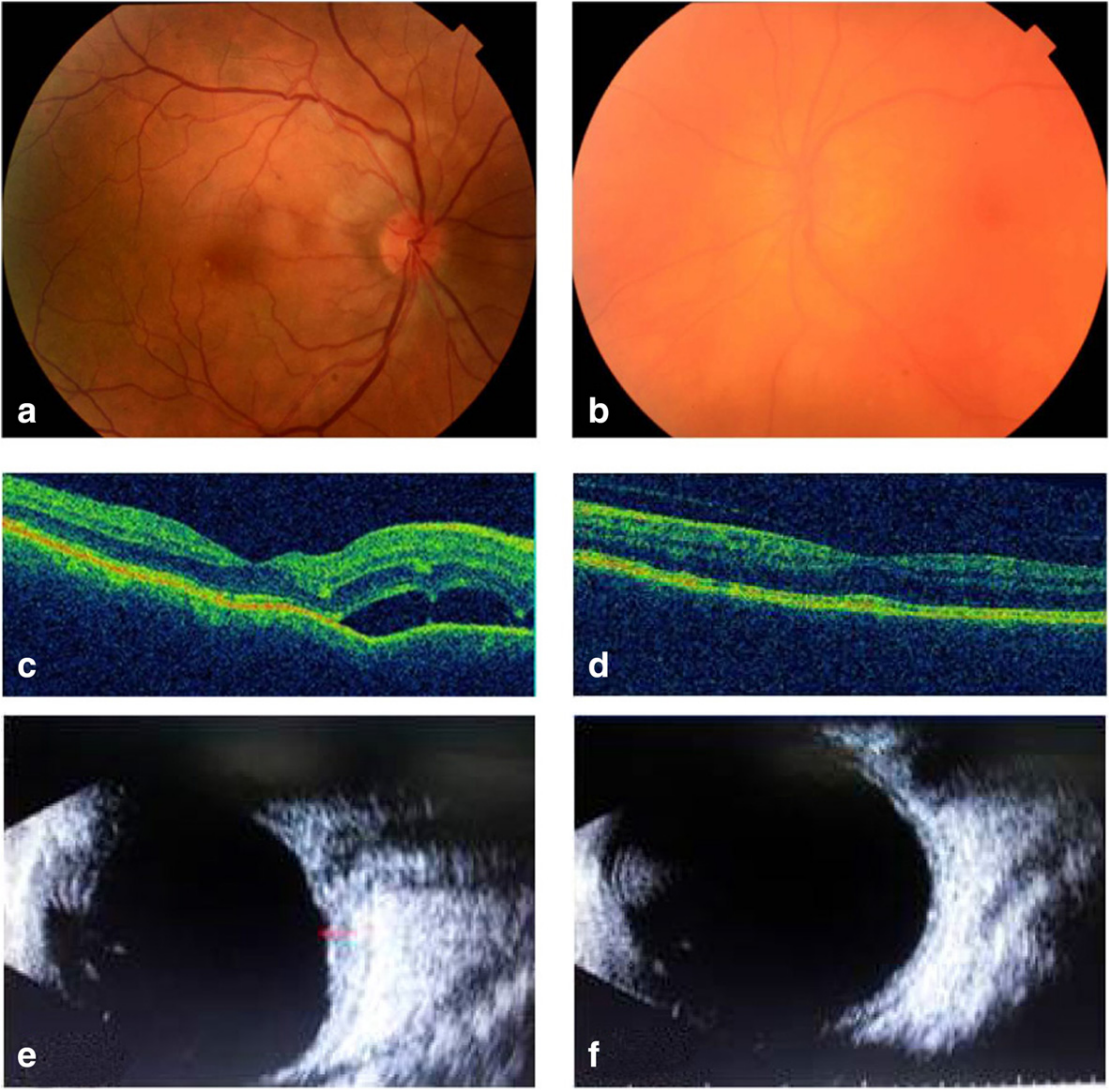
Color fundus photography, optical coherence tomography, and ultrasonography at presentation. **a** Fundus photograph of the right eye at presentation shows optic disc hyperemia and peripapillary exudative retinal detachment. **b** Fundus photograph of the left eye shows +2 vitreous haze and sunset-glow fundus. **c**, **d** Optical coherence tomography shows exudative retinal detachment with a few subretinal septa and retinal pigment epithelium undulations in the right eye and normal findings in the left eye. **e**, **f** B-scan ultrasonography shows a medium-reflective choroidal thickening mostly around the optic disc, in the right eye, and a normal choroidal thickness in the left eye

Best corrected visual acuity (BCVA) was 20/32 in the right eye (RE) and 20/100 in the left eye (LE). Slit-lamp examination showed no anterior chamber inflammation in the RE, but noted few keratic precipitates, +1 cells in the anterior chamber, and localized posterior synechiae in the LE. Ocular pressure was 14 mmHg in both eyes. There were +1 vitreous cells in both eyes and 2+ vitreous haze in the LE. Fundus examination revealed optic disc hyperemia and a shallow ERD in the posterior pole in the RE and diffuse depigmentation producing a sunset-glow appearance with some pigmentary changes in the LE (Fig. 1).

Spectral-domain optical coherence tomography (SD-OCT) showed ERD with a few subretinal septa and undulations of the retinal pigment epithelium (RPE) in the RE. Findings were unremarkable in the LE (Fig. 1).

**Fig. 2:**
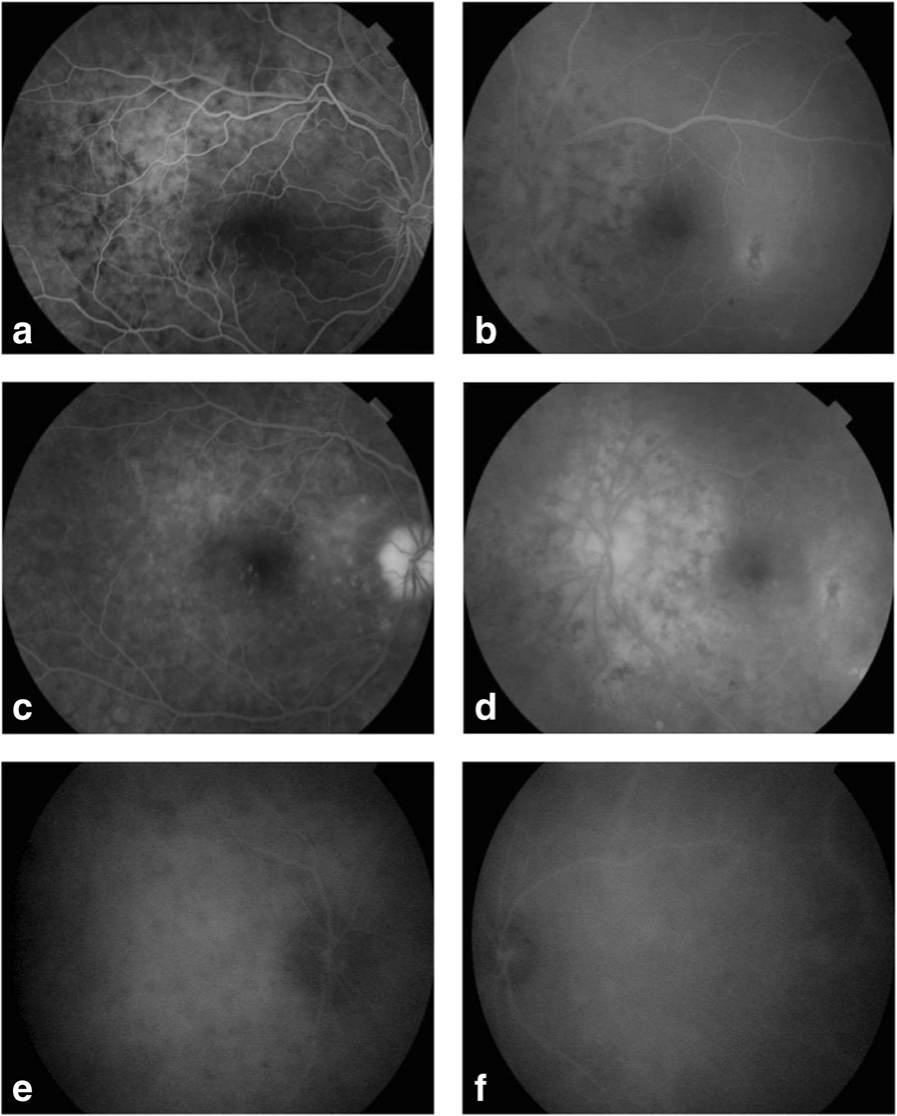
Fluorescein angiography and indocyanine green angiography at presentation. **a** Early-phase fluorescein angiogram of the right eye reveals multifocal areas of delayed choroidal perfusion. **c** Late-phase fluorescein angiogram of the right eye shows multiple areas of pinpoint leakage and optic disc hyperfluorescence. **b**, **d** Fluorescein angiogram of the left eye shows areas of retinal pigment epithelial changes mainly around the optic disc. **e**, **f** Intermediate-phase indocyanine green angiogram shows numerous hypofluorescent dark dots in the right eye and no obvious abnormalities in the left eye

Fluorescein angiography findings included multifocal delayed choroidal perfusion, multiple areas of pinpoint leakage, and optic disc hyperfluorescence in the RE and areas of RPE changes that were more prominent around the optic disc in the LE (Fig. 2). Indocyanine green angiography revealed hypofluorescent dark dots in intermediate and late phases in the RE and showed no obvious abnormalities in the LE (Fig. 2). B-scan ultrasonography showed diffuse medium-reflective choroidal thickening predominating around the optic nerve head in the RE and a normal choroidal thickness in the LE. The axial length was 23.1 mm in the RE and 23.15 mm in the LE (Fig. 1).

Medical history excluded ocular trauma or surgery. Findings in each eye apart were very suggestive of VKH disease, in its acute phase in the RE and in its chronic phase in the LE. Such asymmetry being atypical, a workup was ordered to rule out conditions mimicking VKH disease. Results of systemic examination by the internist, complete blood cell count, sedimentation rate, C-reactive protein, syphilis serology, skin tuberculin test, chest X-ray, and oculo-cerebral magnetic resonance imaging were unremarkable.

**Fig. 3:**
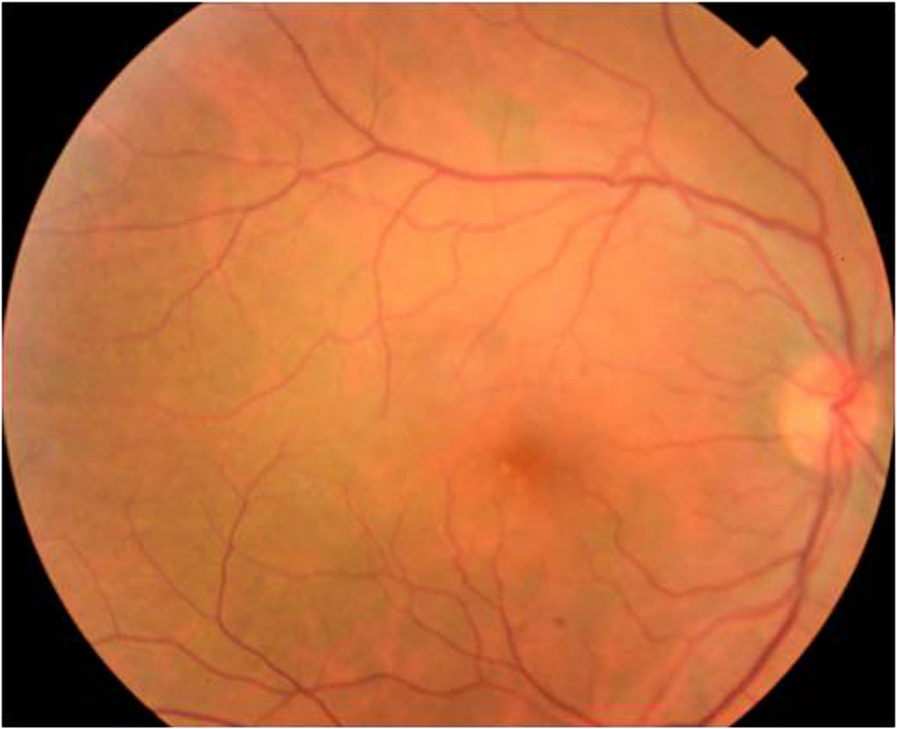
Right color fundus photography at the last follow-up. Fundus photograph of the right eye, 15 months after presentation, shows normal findings

**Fig. 4:**
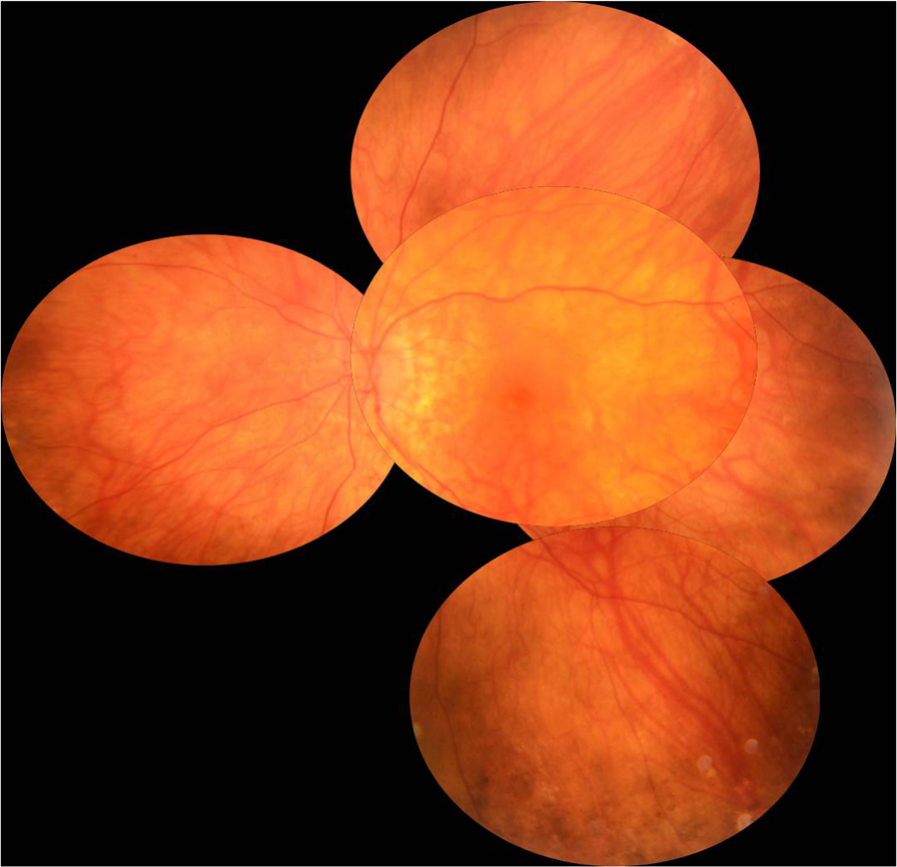
Left color fundus photography at the last follow-up. Composite fundus photograph of the left eye, 15 months after presentation, shows a sunset-glow fundus with retinal pigment epithelial changes and a few peripheral nummular atrophic lesions

The patient was given oral prednisone initiated with a dose of 1 mg/kg/day and then gradually tapered over 6 months. In addition to that, she received mycophenolate mofetil (2 mg a day) for a 12-month duration. After a 15-month follow-up, the patient was still receiving 5 mg of prednisone a day. BCVA was 20/20 in the RE and 20/25 in the LE. Right fundus was normal (Fig. 3). There was a sunset-glow fundus in the LE associated with RPE changes and a few peripheral nummular atrophic lesions (Fig. 4). There were no recurrences or development of integumentary manifestations. Ocular involvement was isolated in our patient, and therefore, a diagnosis of probable VKH disease was made according to the Revised Diagnostic Criteria for VKH disease [1].

This case report was approved by the ethics committee of Fattouma Bourguiba University Hospital, Monastir, Tunisia.

### Discussion

Our patient had an unusual presentation of VKH disease with features of acute disease in one eye and chronic disease in the fellow eye. Other causes of ocular inflammation including malignant hemopathies or other neoplasms, syphilis, tuberculosis, or sarcoidosis were excluded in our patient. The diagnosis of posterior scleritis could be ruled out based on the lack of ocular pain and clinical and ultrasonographic findings.

VKH disease is usually characterized by simultaneous onset of inflammation in both eyes, although the involvement of the second eye may occur 2 to 4 weeks after the first eye [1–3]. There have been very rare cases of VKH disease that remained unilateral after a long follow-up period. However, subclinical disease in the fellow eye was not ruled out in these patients by indocyanine green angiography or B-scan ultrasonography, prior to the initiation of systemic corticosteroids [4]. Unilateral features of acute VKH disease have also been described in a 4-year-old boy. However, ultrasonography in this case revealed choroidal thickening in both eyes, which was consistent with bilateral involvement [5]. A long delay between the first and second eye involvement, ranging from 11 months to 6 years, has been previously reported in four patients with a history of unilateral panuveitis before the definitive diagnosis of VKH disease was established. Baseline subclinical inflammation of the initially unaffected eye might have been overlooked in these cases [6, 7]. On the other hand, prolonged initial unilateral ocular involvement in VKH disease may be misdiagnosed as posterior scleritis [8]. Among these patients with a long interval before the second eye was affected, concomitant sunset-glow fundus in one side and ERD consistent with acute VKH disease in the other side has been noted in only two patients [6, 7].

To the best of our knowledge, our report is the first to describe concurrent acute VKH in one eye and sunset-glow fundus in the fellow eye at initial presentation. This striking contrast between the right and the left eyes might suggest a significant delay before the second eye was affected. However, our patient did not report any ocular complaints when the first eye was affected. She presented only when vision blurring became bilateral. The mechanism of delayed involvement of the second eye remains unclear. Early bilateral involvement with minimal inflammation in the RE seems unlikely in our patient. In fact, without any treatment, sunset-glow fundus would have developed in both eyes.

In summary, patients with VKH disease may present with atypical features of chronic disease in one eye and acute disease in the fellow eye. Initial unilateral ocular inflammation, progressing relentlessly to chronic disease, may remain asymptomatic until the fellow eye is involved. Accurate diagnosis is essential for prompt initiation of corticosteroid and immunosuppressive therapy to reduce the risk of visual morbidity.

## Abbreviations

BCVA: best corrected visual acuity
ERD: exudative retinal detachment
LE: left eye
RE: right eye
RPE: retinal pigment epithelium
SD-OCT: spectral-domain optical coherence tomography
VKH: Vogt-Koyanagi-Harada
